# Ocular Manifestations of the* NAA10*-Related Syndrome

**DOI:** 10.1155/2019/8492965

**Published:** 2019-04-08

**Authors:** Angela S. Gupta, Hind Al Saif, Jennifer M. Lent, Natario L. Couser

**Affiliations:** ^1^Virginia Commonwealth University School of Medicine, Richmond, VA, USA; ^2^Department of Human and Molecular Genetics, Division of Clinical Genetics, Virginia Commonwealth University School of Medicine, Richmond, VA, USA; ^3^Department of Ophthalmology, Virginia Commonwealth University School of Medicine, Richmond, VA, USA; ^4^Department of Pediatrics, Virginia Commonwealth University School of Medicine, Richmond, VA, USA

## Abstract

The* NAA10*-related syndrome is a rare X-linked neurodevelopmental condition that was first described in 2011. The disorder is caused by pathogenic variants in the* NAA10 *gene located on chromosome X at position Xq28. Clinical features typically include severe psychomotor developmental delay, cardiac disease, dysmorphic features, postnatal growth failure, and hypotonia, although there is significant variability in the severity of the phenotype among affected individuals. We describe a 5-year-old female with the syndrome; massively parallel exome sequencing and analysis revealed the c.247C>T (p.Arg83Cys) pathogenic variant that has been previously reported in ten affected individuals. Ocular manifestations of the* NAA10*-related syndrome are not uncommon, although they have not been well characterized in literature reports. From a systematic review of previously published cases to date, ocular abnormalities are present in more than half of patients with the syndrome. Common ocular findings reported include astigmatism, hyperopia, cortical vision impairment, microphthalmia/anophthalmia, and hypertelorism. Our patient presented with growth restriction, dysmorphic features, and hypotonia. Ocular manifestations identified in this child include downslanting palpebral fissures, myopic astigmatism, nystagmus, and exotropia. We speculate that the type and severity of ocular defects present in individuals with the* NAA10*-related syndrome are dependent on the specific* NAA10* pathogenic variant involved.

## 1. Introduction

 Ogden syndrome (MIM #300855) is a rare X-linked neurodevelopmental disorder caused by pathogenic variants of the* NAA10* gene located at Xq28 [1]. The syndrome was originally described in 8 severely affected young boys in two unrelated families by Rope et al. in 2011 [1]. Since the initial report, this description of Ogden syndrome has broadened to include a variety of phenotypes categorized as* NAA10*-related syndrome [2]. Patients have a variety of clinical findings that include postnatal growth failure, delayed psychomotor development, dysmorphic features, and hypotonia. This categorization of* NAA10*-related syndrome also includes Lenz microphthalmia syndrome (MIM#309800), a disorder also caused by pathogenic variants in the* NAA10 *gene which is characterized by abnormalities of the skeletal and urinary systems, teeth, ears, digits, and several ocular defects that may include unilateral or bilateral microphthalmia/anophthalmia, cataracts, nystagmus, coloboma, and glaucoma [3]. A milder* NAA10*-related intellectual disability phenotype associated with different variants has also been described.* NAA10* encodes for the ubiquitously expressed primary protein acetyltransferase in humans, N-terminal-acetyltransferase A (NatA). Thus, a variant and subsequent dysfunctionality of NatA results in profound consequences to the proteome. Thirty-nine individuals with* NAA10 *pathogenic variants with related phenotypic features have been reported [3–10].

Ocular manifestations of the* NAA10*-related syndrome have not been well characterized in the literature. In this report, we summarize the ocular features of the syndrome from a systematic review of the literature and additionally present a young female with the syndrome who initially presented with growth restriction, failure to thrive, and hypotonia. Her ocular manifestations identified include downslanting palpebral fissures, myopic astigmatism, nystagmus, and exotropia.

## 2. Case Report

 The patient, now a 5-year-old female, had a gestation period notable for intrauterine growth restriction (IUGR); her G3P2 mother had pre-eclampsia and hyperemesis during the pregnancy. The patient was born to non-consanguineous parents at 38 weeks by cesarean section, weighing 2.49 kg. She required resuscitation at birth. During infancy, she had hypotonia, laryngomalacia requiring supplemental oxygen, aspiration episodes requiring Nissen and g-tube placement, and prolonged growth failure. Her head circumference maintained trajectory at the 50^th^ percentile, although her length/height has been consistently below the 5^th^ percentile. Her dysmorphic features included broad forehead, midface hypoplasia with prognathism, depressed nasal bridge, hypertelorism, synophrys, deep set eyes, downslanting palpebral fissures, tongue protrusion, occipital flattening, and small hands. 

MRI at age 3 showed ventricular prominence without hydrocephalus and diminutive geni and corpus callosum. EEG showed moderate generalized slowing and occasional independent left and right lateralized slow waves during sleep (bihemispheric dysfunction) and no epileptiform activity. EKG and echocardiogram were normal. 

**Figure 1:**
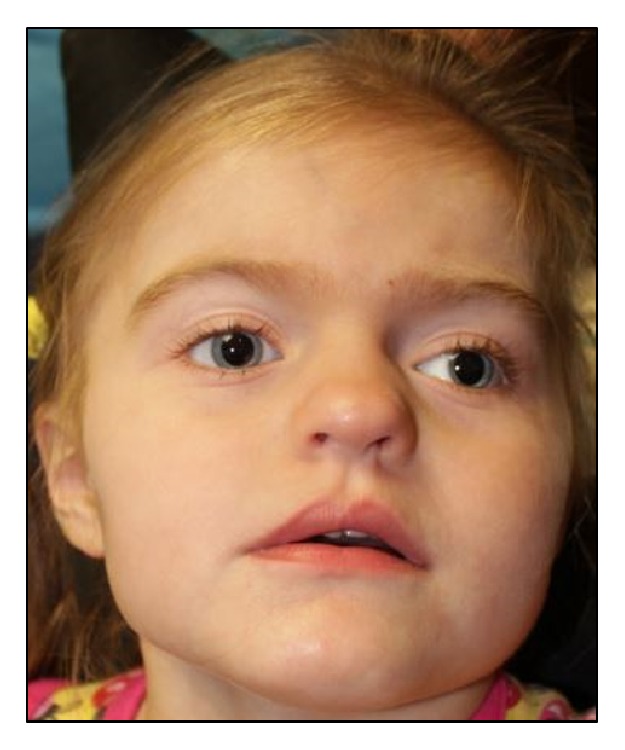
The* NAA10*-related syndrome. A 5-year-old female with a broad forehead, hypertelorism, downslanting palpebral fissures, exotropia, midface hypoplasia with prognathism, and a depressed nasal bridge.

At her last examination at 5 years of age (Figure 1), she remains significantly delayed. She smiles and knows 3-5 single words that are used infrequently. She is able to sit, roll, and start to cruise when placed in standing position. Her ocular abnormalities include having a myopic astigmatism in both eyes requiring glasses, an intermittent alternating exotropia, and high frequency, low amplitude horizontal nystagmus. She was unable to cooperate with eye chart testing, but was able to fix and follow an object with each eye.

Her additional medical problems include idiopathic hypertension, precocious puberty, obstructive sleep apnea, eosinophilic gastritis, seizures, hypohydrosis with overheating, recurrent fever of unknown origin, and intellectual and motor disability. Whole exome sequencing conducted on both parents and the patient (trio WES) identified a pathogenic missense c.247C>T p.Arg83Cys* de novo* variant in the* NAA10* gene. X-inactivation testing was conducted by PCR analysis of a polymorphic CAG repeat in the first exon of the androgen receptor (*AR*) gene. Methylation of sites close to this short tandem repeat has been demonstrated to correlate with X chromosome inactivation [11]. Amplification of the* AR* gene both before and after digestion with the methylation sensitive HpaII restriction enzyme was used to determine the methylation status of the X chromosome. This testing revealed a highly skewed X-inactivation pattern (100:0).

## 3. Methods

We performed a systematic review of the literature to summarize ocular disease in individuals with the* NAA10*-related syndrome. A PubMed/Medline search of “Ogden syndrome” OR “*NAA10*” led us to find a total of 208 articles after removing duplicates on July 2018. No articles were excluded based on year published or language. Articles describing patients with* NAA10 *pathogenic variants with a clinical syndrome consistent with the description by Wu et al. (2018) were included. Within the articles, we identified describing cases of* NAA10-*related syndrome; we reviewed the references to identify other articles that did not appear in our original search.

## 4. Discussion

From our systematic review, we found 9 articles describing 39 reported cases of* NAA10* related syndromes [11]. Including our reported patient, this brings the total number of cases to 40. 50 percent (n=20) of these patients with* NAA10*-related syndrome had ophthalmologic abnormalities (Table 1). The most common ocular findings were astigmatism (n=6), hyperopia (n=4), cortical vision impairment (n=3), hypertelorism (n=3), exotropia (n=3), myopia (n=3), and anophthalmia/micropthalmia (n=3). The most common* NAA10* genetic variant leading to ocular disease, the c.247C>T p.Arg83Cys pathogenetic variant (n=6), results in reduced Acetyl-CoA binding and enzyme activity [5].

The nature of ophthalmologic abnormalities in patients was dependent on the* NAA10* variant involved (Table 1). Rope et al. reported eight patients in two different families with Ogden disease with the c.109T>C p.Ser37Pro variant. No significant visual abnormalities were reported in these patients, although facial dysmorphisms involving the ocular adnexa were present that included hypertelorism, prominent eyes, and downslanting palpebral fissures [1]. Another three patients with a novel missense variant of* NAA10 *(c.128A>C; p.Tyr43Ser) had significant findings on eye exam including moderate right convergent squint, hyperopic astigmatism, and dense right amblyopia [4].

Saunier et al. reported 13 total patients, 9 of which had ocular disease. One patient with a c.319G>T p.Val107Phe variant had astigmatism, strabismus, and mild optic atrophy. Another two patients with the c.384T>A p.Phe128Leu variant had cortical vision impairment. A patient with the c.382T>A p.Phe128Ile variant had hyperopia, astigmatism, exotropia. The remaining five patients had a c.247C>T p.Arg83Cys pathogenic variants similar to our patient; these five individuals displayed a range of clinical findings including myopia, astigmatism, alternating exotropia, cortical vision impairment, and hyperopia.

Three individuals with a severe pathogenic variant in* NAA10* at the intron 7 splice donor site (c.471+2T→A) presented with anophthalmia or microphthalmia, prenatal onset of growth deficiency, and significant dysmorphic features [3].

Valentine et al. described a patient with a c.346C>T, p.Arg116Trp variant with mild ptosis and downslanting palpebral fissures with concurrent absence epilepsy with eyelid myoclonus [5].

Four other studies that reported on individuals with the* NAA10*-related syndrome did not describe any significant ocular findings [6, 7, 9, 10].

Because NAA10 is a ubiquitous protein acetylase, it is not surprising that the phenotype in the* NAA10*-related syndrome involves multiple organ systems, including the eye. The associated ocular abnormalities in the* NAA10*-related syndrome appear dependent on the nature of the pathogenic variant. Patients with more severe ocular phenotypes associated with variants in the* NAA10 *intron 7 splice donor site (c.471+2T>A) described by Esmailpour et al. have a resultant dysgenesis of ocular development associated with the Lenz microphthalmia syndrome. Previous studies inducing knockdown of* NAA10* in zebrafish have resulted in similar abnormalities; zebrafish with* NAA10 *knockdown had anophthalmia or deformed eyes [12]. This could be because splice mutations affect NAA10's ability to interact with other proteins such as TSC2 [3], while missense variants only affect enzyme activity.* NAA10* itself may also play a role in retinoic acid signaling, which is critical for eye development [3]. Further, NAA10 has been reported to acetylate and activate beta-catenin in lung cancer cell lines; and wnt/beta-catenin signaling is known to be crucial for eye development [13]. In the central nervous system specifically, after deletion of* NAA10* myelin basic protein is unstable due to degradation of encephalin, proteins regulating cell survival are affected as well [14, 15]. In the developing brain where* NAA10* is highly expressed, this could be the cause of the cerebral atrophy, cortical vision impairment, and severe neurodevelopmental consequences seen in the* NAA10*-related syndrome.

In summary, this case report and review contributes to our understanding of the relationship of* NAA10* pathogenic variants and ocular abnormalities. Further studies on the role of* NAA10* in eye development can help elucidate the mechanisms underlying ophthalmologic disease in the* NAA10*-related syndrome.

## Figures and Tables

**(a):** 

	Rope, et al. [2011]					Esmailpour, et al. [2014]			Casey, et al. [2015]

*NAA10* gene pathogenic variant	c.109T>C p.Ser37Pro	c.109T>C p.Ser37Pro	c.109T>C p.Ser37Pro	c.109T>C p.Ser37Pro	c.109T>C p.Ser37Pro	Intron 7 splice c.471+2T→A	Intron 7 splice c.471+2T→A	Intron 7 splice c.471+2T→A	c.128A>C p.(Tyr43Ser)

Inheritance	inherited	inherited	inherited	inherited	inherited	inherited	inherited	inherited	inherited

Gender	M	M	M	M	M	M	M	M	M

Eye findings	Hypertelorism	Prominent eyes, downslanted palpebral fissures, ocular hypertelorism	Prominent eyes, downslanted palpebral fissures, ocular hypertelorism	Large eyes, bilateral ptosis	Mild lagophthalmos, infraorbital creases	Anophthalmia/ microphthalmia	Anophthalmia/ microphthalmia	Anophthalmia/ microphthalmia	Moderate right convergent squint, hyperopic astigmatism, dense right amblyopia

Dysmorphic features	+	+	+	+		+	+	+	+

Cardiac anomalies	+	+	+		+				+

Renal anomalies	+	-	-						

Neuro		MRI brain: bilateral globus pallidus T2 prolongation without diffusion restriction, ventricular dilation			Hyper/ hypotonia	MRI- arterial sclerosis	Seizures		Hypotonia. MRI- mild dialation of ventricles nd mild cerebral atrophy

Feeding issues/FTT	-	+	+	+					-

Developmental delay		+		+	+	+	+	+	+

Motor delay		+		+	+				+

**(b):** 

	Saunier, et al. [2016]									Valentine, et al. [2018]	Gupta, et al. [2019]

*NAA10* gene pathogenic variant	c.319G>T p.(Val107Phe)	c.384T>A p.(Phe128Leu)	c.384T>A p.(Phe128Leu)	c.382T>A p.(Phe128Ile)	c.247C>T p.(Arg83Cys)	c.247C>T p.(Arg83Cys)	c.247C>T p.(Arg83Cys)	c.247C>T p.(Arg83Cys)	c.247C>T p.(Arg83Cys)	c.346C>T (p.Arg116Trp)	c.247C>T p.(Arg83Cys)

Inheritance	de novo	de novo	de novo	de novo	de novo	de novo	de novo	de novo	inherited	de novo	de novo

Gender	F	F	F	F	F	F	F	F	F	F	F

Eye findings	Astigmatism, strabismus, mild optic atrophy	Cortical vision impairment	Cortical vision impairment, ambylopia	Hyperopia, astigmatism, exotropia	Myopia, astigmatism	Alternating exotropia, cortical vision impairment	Astigmatism, hyperopia	Hyperopia	Myopia	Ptosis, eyelid myoclonus	Myopic astigmatism, nystagmus, exotropia

Dysmorphic features	+	-				+		+	+	+	+

Cardiac anomalies	+	+	-	+	+	-	+	+	-	-	-

Renal anomalies	-	-	-	-	-	-	+	-	+		-

Neuro	Hypotonia. MRI- thin corpus callosum	Hypotonia. MRI- parenchymal atrophy, thin corpus callosum. Pre and postnatal vetriculomegaly	Hypotonia. Seizures. MRI normal at age 1	Hyper/ hypotonia. MRI- Supraventricular cyst without hyocephalus	Hypotonia. MRI- Periventricular white matter loss	Hyper/ hypotonia. MRI- IVH occipital horn, periventricular leukomalacia, hypoxia ischemic encephalopathy	Hypotonia. MRI normal at age 3		Hypertonia	Hypo/ hypertonia. Absence seizures	Hypotonia, seizures, MRI at age 3- ventricular prominence without hydrocephalus and diminutive geni and corpus callosum

Feeding issues/FTT	+	+	+	+	+	+	+	+		-	+

Developmental delay	+	+	+	+	+	+	+	+	+	+	+

Motor delay	+	+	+		+	+	+	+	+		+
